# Differences in health-related quality of life between the Roma community and the general population in Romania

**DOI:** 10.1186/s41687-022-00530-2

**Published:** 2022-12-22

**Authors:** Tomos Robinson, Yemi Oluboyede, Luke Vale, Elena Olariu

**Affiliations:** Health Economics Group, Population Health Sciences Institute, Newcastle University, Baddiley-Clark Building, Richardson Road, Newcastle Upon Tyne, NE2 4AX UK

**Keywords:** Health-related quality of life, EQ-5D-5L, EQ-VAS, Romania, Roma people

## Abstract

**Background:**

Previous research has shown that Roma people report worse health outcomes than the general population and suffer from a myriad of economic and social disadvantages. The objective of this study was to assess the differences in the health-related quality of life (HRQoL) between the Roma people and the Romanian general population.

**Methods:**

Two cross-sectional surveys were conducted face-to-face in 2018 and 2019 in two nationally representative samples of both the general population and Roma communities, recruited from all regions of Romania. Both samples completed the EQ-5D-5L and EQ-VAS questionnaires, as well as a range of sociodemographic questions. Coarsened Exact Matching and several different regression models were used to assess the differences in HRQoL between the two groups.

**Results:**

2308 respondents were included in the matched sample: 1,621 general population individuals; 687 Roma people. Roma people had more problems with self-care, pain/discomfort, and anxiety/depression than the general population. They also reported a lower overall level of HRQoL than the general population of Romania, as reflected by the lower EQ-5D-5L and EQ-VAS scores. Our sensitivity analysis between Coarsened Exact Matching and other matching procedures showed consistent results across all regression models.

**Conclusions:**

In Romania, the Roma community has a lower level of HRQoL than the general population. Understanding the underlying causes of this inequality should be the focus of future research. Policies aimed at reducing the level of health inequality between the Roma and the general populations should be promoted locally.

**Supplementary Information:**

The online version contains supplementary material available at 10.1186/s41687-022-00530-2.

## Background

The Roma community is one of the largest ethnic minorities in Central, Southern and Eastern Europe [1], the community being characterised by a wide range of different customs and languages. Romania has one of the largest Roma populations in Europe, being the second largest minority in the country [2], with unofficial figures going as high as 10% of the total population [3].

Throughout Europe, the Roma community experience a range of social exclusions relative to the general population, with severe consequences for their health and level of education [4]. The situation is no different in Romania: previous studies have found the Roma to be significantly more likely to report at least one chronic condition [5], to be less likely to complete required education [2], to have a lower level of social health insurance [2] and a shorter life expectancy [6] than non-Roma people. Additionally, Romanian Roma children experience a higher burden of mental health problems than non-Roma children [7]. These gaps between the general population and the Roma will most probably be aggravated in the future as inequalities have increased lately in Romania [8] and will increase even more in Romania, if the current low levels of government expenditure are maintained in the country [9].

The poor health of the Roma people cannot be fully attributed to socioeconomic factors, with ethnicity most probably playing a key role in the observed differences [10]. Even though there is a clear association between health and ethnicity [11], there are only a few studies conducted on this topic in this population [11]. Additionally, the majority of literature available for this vulnerable group has focused on a narrower definition of health, covering topics such as self-rated health, non-communicable disease, or child and adolescent health [12]. Adopting a broader definition of health, such as the one provided by health-related quality of life (HRQoL), allows a better understanding of health problems [13] and personal well-being [14]. Additionally, it can be used to identify where health inequalities exist between groups and where health and social interventions are needed to reduce the level of inequality.

To date, no study has compared the level of HRQoL (as measured by internationally validated instruments, such as the EQ-5D-5L [15]) between the general population and Roma people in Romania. Given this background, the objective of this study was to assess the differences in the HRQoL between the Roma people and the Romanian general population using a combination of matching and regression methods (see “Descriptive analysis” section). Similar methods have been used in previous studies in different contexts, including the comparison of different groups within and between populations [16–18]. We hypothesised that the Roma people would have a lower level of HRQoL than the Romanian general population.

## Methods

### Data and procedure

We used data from two surveys:

i. A general population survey conducted from November 2018 to November 2019 in a national representative sample of non-institutionalized adults (older > 18) who were living in Romania at the time of the study (*general population survey*).
  In the general population survey, face-to-face interviews were conducted by trained interviewers in a national representative sample of adults selected from 32 settlements from all regions in Romania. Interviews were computer-assisted and used a secure online survey site (EQ-VT Software V2.1) developed by the EuroQoL Research Foundation. Settlements were selected randomly after having divided Romania’s territory into strata based on region and settlement size. Households were selected using a random route procedure [19]. Individuals within households were selected using the next birthday rule. More details on the sample design can be found elsewhere [20].
  People were interviewed following a standardized interviewing procedure as agreed in the protocol approved by the EuroQoL Research Foundation. The interview consisted of multiple sections: the EQ-5D-5L questionnaire and background questions on gender, age and experience of illness, valuation tasks for EQ-5D-5L and EQ-5D-3L, the EQ-5D-3L questionnaire and a country-specific questionnaire. The quality of the data was checked bi-weekly as agreed with the EuroQoL Research Foundation. Additionally, telephone contact was made with a random 54.3% of the sample to confirm their address and participation in the study. More details can be found elsewhere [20, 21].
ii. A survey conducted from November 2018 to February 2019 in a national sample of self-declared Roma, older than 18 years old who were living in Romania at the time of the study (*Roma communities survey*).
  In the Roma communities survey, face-to-face interviews were conducted by a survey research company with experience in performing interviews with Roma communities. Interviews were pen and pencil as this was the standard mode of data collection of the contracted survey research company at that time and were performed in approximately the same settlements selected for the general population survey. However, nine settlements were replaced due to the fact that few or no Roma people lived there according to the 2011 census and three settlements were replaced with settlements of similar Roma population size from within the same strata due to improved access to those communities.
  Of all respondents, 20.2% were selected using a random route procedure particularly in those settlements that matched the settlements selected for the general population survey. The remainder of respondents were selected using a snowballing technique [22]: respondents that agreed to participate in the study were asked to recommend one other household with similar characteristics that the interviewer could approach regarding participation in the study. For more details on how the sample was selected, please see Olariu et al. [20].
  Respondents were interviewed by trained interviewers that had previously worked with Roma communities. All respondents were treated with care and sensitivity and social worker assistants facilitated interviewers’ access to communities. Suggestions from members of the public and Roma community health mediators (Roma women trained to liaise between Roma communities and healthcare professionals [23]) were incorporated into the design of the participant information sheets and the survey was pretested with ten Roma volunteers from the city of Cluj-Napoca.
  The interview consisted of presentation of the study, signing of informed consents, the EQ-5D-5L questionnaire and questions related to sociodemographics.
  Quality assurance procedures consisted of phone calls made to 33% of the interviewees to confirm the interview and the selection procedures and face-to-face visits to some additional 16% of the interviewees.

### Measures

#### Outcome measure

HRQoL was measured using the EQ-5D-5L questionnaire [15]. The EQ-5D-5L questionnaire is a short, simple and generic instrument that measures health status using five dimensions: mobility, self-care, usual activities, pain-discomfort, anxiety-depression. It assesses the severity of problems in each dimension using a five-point Likert scale that ranges from ‘no problems’ through ‘slight’ and ‘moderate’ problems to ‘severe’ and ‘extreme’ problems. The EQ-5D-5L is accompanied by a Visual Analog Scale (EQ-VAS) that provides a quantitative measure of overall health status. On the EQ-VAS, respondents are asked to mark their own health on a scale that ranges from 0 (worst imaginable health state) to 100 (best imaginable health state). The answers to the EQ-5D-5L can be converted to utility scores using country specific value sets. A country specific value set for the Romanian version of EQ-5D-5L has recently become available [21].

#### Sociodemographic measures

The country-specific questionnaire used in the general population survey included questions on place of residence, ethnicity, caregiver and parenting status, health literacy, preference over length or quality of life, marital status, education level, religion, employment status and income.

The sociodemographic questionnaire used in the Roma communities survey consisted of the same questions that were used in the general population survey, plus one question on the availability of health mediators in the respective community and one on the respondent’s ability to write or read, as literacy amongst the Roma has been reported to be as low as 61% [2]. Additionally, the question on employment status was adapted to better reflect the particularities of the Roma communities. Specifically, Roma are more likely to be performing elementary occupations, traditionally low skilled with low remuneration [24].

### Data analysis

#### Descriptive analysis

We first performed a descriptive analysis of the sociodemographic characteristics in the Roma communities and general population, with variables summarised using means and standard errors. We also calculated the proportion of respondents reporting any problem in any EQ-5D-5L dimension, as well as the mean EQ-VAS score and EQ-5D-5L utility score for both groups.

#### Matching procedure

In order to estimate the difference in HRQoL between the general population and the Roma communities, we used matching methods, specifically coarsened exact matching (CEM), which has been shown to outperform other matching methods on a variety of aspects [25]. The idea of CEM is to temporarily coarsen each variable included in the matching procedure into substantively meaningful groups, exact match on these coarsened data, and then only retain the original (uncoarsened) values of the matched data [26]. Information on the characteristics collected in the survey that were available and considered for use in the matching process are provided in Table 1.

**Table 1:** Variables considered for use in the empirical analyses

Variable	Variable type	Categories
Age	Continuous	N/A
Gender	Categorical	1 = male; 0 = female
Marital status	Categorical	1 = married or living with partner; 0 = separated, divorced, not living with partner, widow, never married
Number of children	Continuous	N/A
Urbanity	Categorical	1 = urban; 0 = rural
Religion	Categorical	1 = Christian Orthodox; 0 = other religion or no religion
Low education	Categorical	1 = no education, low level of education; 0 = medium/tertiary education
Low income	Categorical	1 = income below national average; 0 = income equal or above national average
Employment	Categorical	1 = employed; 0 = unemployed, retired, domestic, in education, unable to work

To measure the balance in the distributions of characteristics following matching, we used the ${L}_{1}$ statistic to examine balance on the joint distribution of all characteristics [26]. The ${L}_{1}$ statistic can take values from 0 to 1, where 0 denotes perfect balance on all characteristics, and 1 denotes complete separation. The ${L}_{1}$ value is not valuable on its own, and can instead be seen as a point of comparison between the matching solutions. When matching two samples, the ideal value for the ${L}_{1}$ statistic should be as low as possible while maintaining a relatively high proportion of individuals from the original samples [27]. One further advantage of the CEM procedure is that it matches on missing values in the data set, and therefore we did not need to implement missing data strategies such as multiple imputation or inverse probability weighting. For a comprehensive overview of CEM, please see Iacus et al. [25].

Different econometric models can be applied to the dataset after the CEM procedure has been completed, by running a regular regression model that includes a set of sample weights generated from the matching procedure to account for the matching process. All matching analyses were conducted using the *CEM* and *IMBALANCE* commands in Stata version 15 (StataCorp, College Station, TX, USA). To test the results from the CEM models, we performed a sensitivity analysis by running Propensity Score Matching (PSM) models on the restricted data for both the EQ-VAS score and EQ-5D-5L utility scores, as shown in Additional file 1: Appendix 1 and Additional file 2: Appendix 2. In these PSM models, the nearest neighbour matching method with a single match per observation was used and the Average Treatment Effect on the Treated (ATET) calculated. PSM models were run using the *PSMATCH* command.

#### Regression analysis

**Fig. 1:**
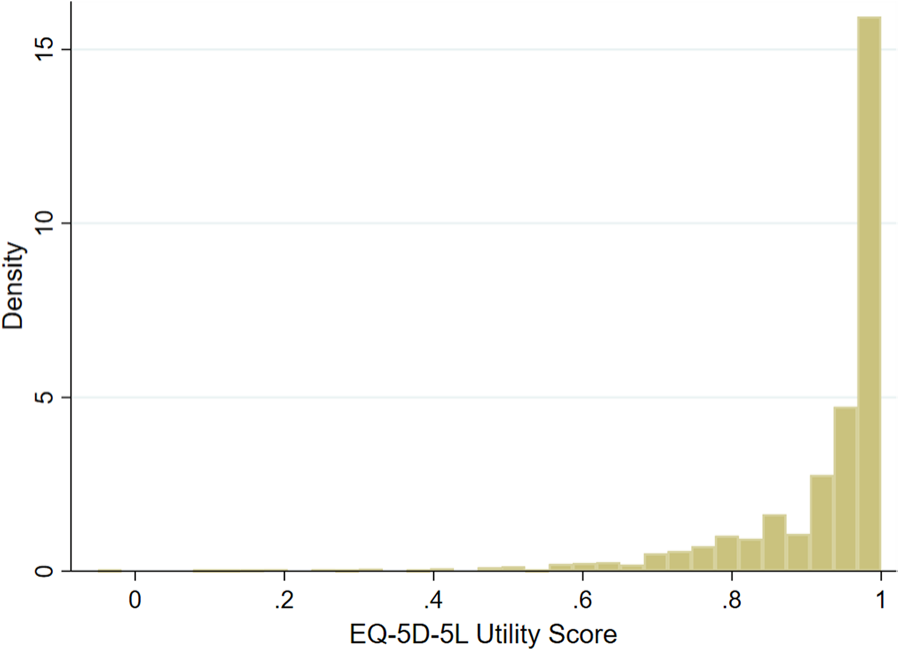
Distribution of EQ-5D-5L utility score

Several econometric models were used according to the nature of the dependent variable:

A. For the individual EQ-5D-5L dimensions, we used a set of Ordered Logit (OLOGIT) models on the matched data. The OLOGIT model was used due to the ordinal nature of the dependent variable in these regression models.
B. For the EQ-VAS score (which is scored on a 0–100 scale), we used an Ordinary Least Squares (OLS) model and a Generalized Linear Model (GLM). The OLS model is the most common starting point in econometric analysis and in this case serves as the base model. Building on this, the GLM was implemented to take account of the non-normality of the distribution of the dependent variable. We used the modified Park Test [28] to determine the correct GLM specification. In this case, a Gaussian model with a log-link was found to be the most appropriate specification.
C. For the EQ-5D-5L utility scores, we used OLS, GLM specifications and Tobit models. In a similar manner to the EQ-VAS regressions, the OLS model served as the base model and the GLM was implemented to take account of the non-normal distribution of the dependent variable. Furthermore, we used a Tobit model to take account of the fact that there were a large number of observations (50.58%) at the highest possible value (1) in the estimation sample (this was expected as these are population samples and many people would be expected to be in good health). The distribution of EQ-5D-5L utility score is shown in Fig. 1*.* The results from the modified Park Test again found that the Gaussian model with a log-link was the most appropriate specification.

For all models, the sample weights generated from the CEM matching procedure were used. Robust standard errors were used in all analyses and regression analyses were conducted using the *OLOGIT, REGRESS, GLM* and *TOBIT* commands in Stata version 15.

## Results

### Sociodemographic characteristics

The full sample size was 2308. It consisted of 1621 individuals from the general population and 687 individuals from the Roma community. Response rates and a list with settlements where interviews were conducted for both surveys can be found in Additional file 1: Appendix 1.

The sociodemographic characteristics of the sample are displayed in Table 2. As expected, the two samples differed in the majority of the characteristics. Compared to the general population, on average the Roma sample were younger, more likely to be married, had a higher number of children, a higher proportion of people with low or no education, more people with income levels below the average and fewer employed persons than the general population sample. In both samples, rural areas and women were under-represented when compared with the available national statistics [29].

**Table 2:** Sociodemographic characteristics of the general population and Roma community samples

Variable	General population Mean (standard error)	Roma community Mean (standard error)	Mean difference (standard error)	N (% of full sample)
Age	48.46 (0.41)	41.98 (0.59)	6.48*** (0.73)	2308 (100%)
Gender				
Female	0.65 (0.01)	0.55 (0.02)	0.10*** (0.01)	2308 (100%)
Marital status				
Married/living with partner	0.66 (0.01)	0.79 (0.02)	0.13*** (0.02)	2308 (100%)
Number of children	1.27 (0.29)	3.05 (0.08)	1.78*** (0.07)	2293 (99.35%)
Urbanity				
Reside in urban area	0.74 (0.01)	0.66 (0.02)	0.08*** (0.02)	2308 (100%)
Religion				
Christian-Orthodox	0.85 (0.01)	0.79 (0.02)	0.06*** (0.02)	2269 (98.31%)
Low education				
No or low level of education	0.12 (0.01)	0.69 (0.02)	0.57*** (0.02)	2305 (99.87%)
Low income				
Income below national average	0.46 (0.01)	0.97 (0.01)	0.51*** (0.02)	2064 (89.43%)
Employment				
Employed	0.61 (0.01)	0.50 (0.02)	0.11*** (0.02)	2132 (92.37%)

Standard errors in parentheses

***p < 0.0

As shown in Table 3, on average the Roma community also reported a worse level of HRQoL than the general population. For example, they were less likely to report ‘no problems’ for all individual EQ-5D-5L dimensions aside from the ‘Mobility’ dimension. Moreover, the Roma community on average were less likely to report the best health state from the EQ-5D-5L classification system (11111) and had a lower HRQoL score on average as measured by the EQ-5D-5L utility score and EQ-VAS score. These patterns were further explored in the regression analyses.

**Table 3:** Health-related quality of life of the general population and Roma community samples in the full sample (N = 2308)

	General population Mean (standard error)	Roma community Mean (standard error)	Mean difference (Standard error)	N (% of full sample)
*EQ-5D-5L dimensions*				
Mobility				
No problems	0.78 (0.01)	0.76 (0.02)	0.02 (0.02)	2,307 (99.96%)
Any problems	0.22 (0.01)	0.24 (0.02)		
Self-care				
No problems	0.90 (0.01)	0.79 (0.02)	0.11*** (0.02)	2,307 (99.96%)
Any problems	0.10 (0.01)	0.21 (0.02)		
Usual activities				
No problems	0.82 (0.01)	0.75 (0.02)	0.07*** (0.02)	2308 (100%)
No problems	0.18 (0.01)	0.25 (0.02)		
Pain/discomfort				
No pain	0.63 (0.01)	0.51 (0.02)	0.12*** (0.02)	2303 (99.78%)
Any pain	0.37 (0.01)	0.49 (0.02)		
Anxiety/depression				
Not anxious or depressed	0.77 (0.01)	0.62 (0.02)	0.15*** (0.02)	2288 (99.35%)
Any anxiety or depression	0.23 (0.01)	0.38 (0.02)		
EQ-5D-5L utility				
11111	0.53 (0.01)	0.45 (0.02)	0.08*** (0.02)	2285 (99.00%)
Utility score	0.94 (0.01)	0.89 (0.01)	0.04*** (0.01)	2,285 (99.00%)
EQ-VAS	82.71 (0.38)	74.72 (0.82)	7.98*** (0.79)	2308 (100%)

Robust standard errors in parentheses

***p < 0.01

### Missing data

Overall, the level of missing data was low in both samples. The general population sample had no missing values in any of the outcome variables (EQ-VAS, EQ-5D-5L utility scores or EQ-5D dimensions) as the valuation tasks of the survey had a hard choice format, meaning the interviewer could not proceed to the following question unless an answer was recorded for the currently displayed question. In the Roma community sample, there were relatively little missing data for the EQ-5D-5L individual dimensions and EQ-5L-5L utility scores (see Additional file 2: Appendix 2). However, there were substantial levels of missing data in some of the variables considered for the CEM procedure, such as employment and income (see Table 4).

**Table 4:** Summary statistics for the main variables considered in the CEM procedure for the two samples and the percentage of missing values for each of them

Characteristic	General Population (n = 1621)	% Missing	Roma Community (n = 687)	% Missing	Difference
Age	48.461	0	41.981	0	6.480
Male	0.352	0	0.451	0	0.101
Married	0.658	0	0.792	0	0.134
Urban	0.741	0	0.657	0	0.084
Number of children	1.271	0.93	3.052	0	1.781
Orthodox Christian	0.852	1.05	0.788	3.20	0.064
Low education	0.885	0.12	0.310	0.15	0.574
Employed	0.613	2.53	0.504	19.65	0.109
Low Income	0.425	7.71	0.970	17.32	0.545

### Balancing

As previously noted, the two samples were severely imbalanced in terms of age, number of children, proportion of individuals reporting being on a low income and proportion of individuals reporting having a low level of education. When these variables were included in the CEM procedure, only 254 individuals were matched between the two samples: 177 general population individuals and 77 Roma individuals. Consequently, the income and number of children variables were ultimately not included in the final matching procedure, and the age variable was coarsened into five age categories (18–30; 30–40; 40–50; 50–60; 60+) and matched on these categories rather than being matched on the continuous variable.

Tables 5 and 6 show the imbalance in the distributions of variables before and after the CEM procedure. Implementing CEM resulted in 347 strata, of which 109 contained both general population and Roma respondents. The sample size following matching was 1612, 70% of the original sample size of 2308. As expected, there was a reduction in imbalance following matching, with the L1 statistic falling from 0.709 to 0.425 (values closer to 0 denote perfect balance). As seen in Table 6, following the matching procedure the distributional characteristics of the covariates are identical for the general population and Roma respondents, with the notable exception of the age variable. In order to account for the remaining differences in age, we additionally controlled for the continuous age variable in all regression models.

**Table 5:** Imbalance in the distributions of variables in the raw data

Characteristic	Difference in mean	Difference in minimum	Difference in 25th percentile	Difference in 50th percentile	Difference in 75th percentile	Difference in maximum
Age	− 6.625	0	− 9	− 8	− 7	− 7
Male	− 0.105	0	0	0	0	0
Marital status	0.129	0	1	0	0	0
Urban	− 0.080	0	0	0	0	0
Orthodox Christian	− 0.050	0	0	0	0	0
Low education	− 0.525	0	− 1	− 1	0	0
Employed	− 0.112	0	0	− 1	0	0

Overall L1 statistic = 0.709

**Table 6:** Imbalance in the distributions of variables following matching

Characteristic	Difference in mean	Difference in minimum	Difference in 25th percentile	Difference in 50th percentile	Difference in 75th percentile	Difference in maximum
Age	− 1.18	0	− 1	0	− 1	− 3
Gender	0	0	0	0	0	0
Marital status	0	0	0	0	0	0
Urban	0	0	0	0	0	0
Orthodox Christian	0	0	0	0	0	0
Low education	0	0	0	0	0	0
Employed	0	0	0	0	0	0

Overall L1 statistic = 0.425

### Differences in HRQoL between general population and Roma respondents

#### EQ-5D-5L dimensions

Table 7 shows the results from the Ordered Logit regression models following the matching procedure. As shown, across all EQ-5D-5L dimensions, the Roma population were more likely to report more problems than the general population. For example, the odds of having problems with Self-Care were 2.84 times higher in the Roma population than in the general population. Additionally, the odds of suffering from depression/anxiety or from pain/discomfort were 2.158 higher and 2.366 higher in the Roma people as compared to the general population respectively. Differences in the M*obility* (1.222) and U*sual Activities* (1.299) dimensions between the two groups were smaller in magnitude and associated with a higher level of uncertainty.

**Table 7:** Differences in EQ-5D-5L individual dimensions between the general population and Roma respondents (n = 1612)

EQ-5D-5L dimension	Roma	Lower 95% CI	Upper 95% CI
Self-care	2.840*** (0.929)	1.496	5.392
Mobility	1.222 (0.304)	0.751	1.991
Usual activities	1.299 (0.320)	0.801	2.103
Pain and discomfort	2.366*** (0.474)	1.600	3.505
Anxiety and depression	2.158*** (0.393)	1.389	3.355

Odds ratios from ordered logit models. All models include CEM weights and are additionally adjusted for age. Robust standard errors in parentheses

***p < 0.01

#### EQ-VAS

Table 8 shows the results from the OLS models and GLMs following the matching procedure. As shown, on average the Roma community reported an EQ-VAS score 8.001 lower in the OLS model and 7.629 lower in the GLM. This general pattern of results was replicated in the PSM model, where the Roma community were estimated to report an EQ-VAS score 6.437 lower than the general population (see Additional file 3: Appendix 3).

**Table 8:** Differences in EQ-5D-5L utility and EQ-VAS scores between the general population and Roma respondents

	Roma	Lower 95% CI	Upper 95% CI
**Unadjusted differences (full sample: N = 2308)**			
EQ-5D-5L utility	− 0.043*** (0.006)	− 0.032	− 0.055
EQ-VAS score	− 7.978*** (0.791)	− 9.529	− 6.427

Regression modelling			
Model	Roma coefficient	Lower 95% CI	Upper 95% CI
**EQ-5D-5L utility (matched sample: N = 1612)**			
OLS	− 0.046*** (0.009)	− 0.065	− 0.028
GLM	− 0.044*** (0.009)	− 0.062	− 0.026
Tobit	− 0.054*** (0.017)	− 0.098	− 0.030
**EQ-VAS score (matched sample: N = 1612)**			
OLS	− 8.001*** (1.382)	− 10.712	− 5.289
GLM	− 7.629*** (1.428)	− 10.427	− 4.830

For regression models, coefficients are marginal effects from Ordinary Least Squares Models, Generalized Linear Models with a Gaussian Distribution and a Log Link Function and a Tobit Model right censored at 1. All models include CEM weights and are additionally adjusted for Age. Robust standard errors in parentheses

***p < 0.01

#### EQ-5D-5L utility

As shown in Table 8, on average the Roma population also reported an EQ-5D-5L utility score lower than that of the general population. The magnitude of these differences were 0.046 and 0.044 in the OLS model and GLM respectively. These results were also similar in magnitude to those from the PSM model (-0.040) (see Additional file 4: Appendix 4). The magnitude of the difference was marginally higher in the Tobit model, with the Roma population reporting an EQ-5D-5L utility score 0.054 lower than the general population on average.

## Discussion

Our results show that, on average, the Roma community report a lower level of HRQoL than the general population in Romania. This finding was consistent across individual EQ-5D-5L dimensions, the EQ-VAS and the EQ-5D-5L utility score. These results are in line with previous studies that have also shown that Gypsies and Travelers (English Gypsies, Welsh Gypsies, Scottish Gypsy Travelers or Irish Travelers),as described by the study authors, have a poorer health status (as measured with EQ-5D) than those in low socioeconomic groups in the UK [30] and that their EQ-5D utility scores are significantly lower than those of the general population [31]. Additionally, a similar drop in HRQoL, as measured in our study by the EQ-VAS, has also been reported in other studies that compared the health status of Gypsies and Travelers from Sheffield (English Gypsies, Welsh Gypsies, Irish Travelers) with that of a UK resident non-Traveler sample matched for age and sex [32]. Finally, our high completion rates of the EQ-5D-5L in the Roma community sample have shown that using EQ-5D-5L as a measure of HRQoL in face-to-face interviews is feasible in this vulnerable group. This is in line with findings from other studies conducted in the UK in this population [30]. These results are encouraging, as they imply that the EQ-5D-5L can be used in this community in future empirical studies, for example when looking at the effectiveness of health interventions designed to improve the health and well-being of the Roma people.

If we were to convert our results into quality adjusted life years (QALYs), there would be a difference of approximately four QALYs between the two groups, assuming the average life expectancy in Romania (75 years) for both groups and the average EQ-5D-5L utility scores for each group. This difference would be even larger if we were to take into account that the average life expectancy of the Roma people can be up to a decade shorter than that of the general population [33].

The drivers of poorer HRQoL in Roma people were problems with self-care, anxiety and depression and pain and discomfort. These results are in line with those found by Parry et al. [32] that showed that the Roma people are more likely to experience pain or discomfort and anxiety or depression (as measured with EQ-5D) than a sample of the general population in the UK. Other studies have also underlined that the Roma are at higher risk of mental health problems [7, 34, 35] due to ongoing discrimination and social exclusion [36]. Problems with mobility and usual activities were not significantly associated with a lower HRQoL in Roma people in our study. This is in contrast with other studies conducted in the general population in Romania that showed that pain and discomfort and mobility have the highest negative impact on HRQoL in Romania [10, 21]. This difference might be due to how the Roma perceive health and their beliefs that health problems are usually associated with pain and unhappiness [37].

To match our two samples, we used CEM. We chose CEM over other matching procedures, such as PSM, as it has the ability to achieve lower levels of imbalance, model dependence and bias than PSM [38] and outperforms other matching methods (for instance PSM) on a variety of other criteria [26]. Additionally, it is computationally very efficient even for large datasets [26] and it can match on missingness or work well with multiple imputation methods for missing data [26]. However, some authors have argued that matching procedures such as CEM tend to reduce the original sample size and thus potentially increase bias (as well as imprecision) [39]. Additionally, model dependence has been reported to increase significantly when the number of additional covariates is increased [40], with some recommending limiting covariates to fewer than ten [41]. In this study, we used seven covariates to balance our samples and retained 70% of the original sample size.

### Strengths and limitations

There are several strengths to this study. Firstly, both surveys were conducted in all regions of Romania and the same questionnaire, recognized internationally to be valid and reliable [42–44], the EQ-5D-5L, was applied to both groups. We also managed to recruit successfully comparatively large samples in both populations, succeeding in including in relatively high numbers a marginalized and often hard to reach population in research (Roma people). Thirdly, we had very low levels of missing data in both surveys and especially for our main outcome, the EQ-5D-5L. Finally, our sensitivity analysis regarding the matching procedure returned models that showed consistent results.

However, there are some associated weaknesses that should be acknowledged. Firstly, our study consisted of self-identified Roma adults. Several studies in Romania have shown that self-identification (an individual’s private conception of their ethnicity[45]) and hetero-identification (the ethnicity attributed to an individual by an observer [45]) generally do not overlap [46]. Additionally, self-identification is more common in compact Roma communities than in mixed communities, with the first being characterized by a higher rate of poverty [47]. Moreover, the majority of our Roma sample was recruited using a snowball technique with respondents being asked to refer one other household of similar characteristics that might be interested in participating in the study. Hence, our sample might consist mainly of Roma people with similar traits and living conditions and therefore, our results might overestimate the difference between the Roma people and the general population through the inclusion of Roma respondents mainly from more socially disadvantaged communities. However, the use of a relatively large sample should have partially addressed this potential selection bias. Secondly, we used the 2011 census to decide if interviewing Roma people in all settlements selected for the general population study was feasible or not. At present, there is widespread agreement in the literature that a high number of people might not declare themselves as Roma in official contexts, such as the census, due to stigma associated with Roma identity [45]. Hence, census data in many Eastern European countries tends to underestimate the size of the Roma population [45]. However, at present in Romania there is no agreed-upon sampling frame for Roma samples and using census data was our best available option given our budget and time constraints. Thirdly, the questionnaire completed by the Roma community was pen and pencil, whereas the questionnaire completed by the general population sample was computer assisted. It is possible that this difference may have impacted the responses to the HRQoL and sociodemographic questions. However, PROMs (patient reported outcome measures) administered on paper, like EQ-5D, have been shown to be comparable to measures administered on an electronic device according to a systematic review conducted by Muehlhausen et al. [48]. Finally, the higher female participation in our study might affect its representativeness. Fewer men than women were available to interview especially in rural areas for both surveys. This might be related to the temporary migration for work that has been increased in Romania in the past few years and has attracted especially men from rural areas [49].

## Conclusions

In conclusion, in this study we found that levels of HRQoL (as measured by the EQ-5D-5L) are lower in the Roma community than the Romanian general population. Although it is well known that the Roma community suffer from a range of social disadvantages, further research, potentially through qualitative studies, is required to truly understand the underlying mechanisms of the apparent inequalities in health and well-being displayed in this study and so identify and remove potential barriers that Roma face in accessing good quality health care.

## Supplementary Information

**Additional file 1.** Appendix 1 – Refusal rates and settlements and where interviews took place for the general population survey and Roma communities survey.

**Additional file 2.** Appendix 2 – Missing data in the EQ-5D-5L measures for the Roma community (*n* = 687).

**Additional file 3.** Appendix 3 - Differences in EQ-VAS scores between the general population and Roma respondents from Propensity Score Matching Models (*n* = 1602).

**Additional file 4.** Appendix 4 - Differences in EQ-5D-5L utility scores between the general population and Roma respondents from Propensity Score Matching Models (*n* = 1589).

## Data Availability

The data used for this manuscript are available from the corresponding author upon reasonable request.

## Abbreviations

CEM: Coarsened Exact matching
EQ-VAS: Visual Analog Scale
GLM: Generalized Linear Model
HRQoL: Health-related quality of life
OLS: Ordinary Least Squares
PSM: Propensity Score Matching
QALY: Quality adjusted life year
