# Supplementary material for: Differences in health-related quality of life between the Roma community and the general population in Romania
Source: J Patient Rep Outcomes. 2022 Dec 22;6:127. doi: 10.1186/s41687-022-00530-2 (PMC9780407; doi:10.1186/s41687-022-00530-2)
Supplement: Supplementary file 1 — Additional file 1. Appendix 1 – Refusal rates and settlements and where interviews took place for the general population survey and Roma communities survey. [file 41687_2022_530_MOESM1_ESM.docx]

Appendix 1 – Refusal rates and settlements and where interviews took place for the general population survey and Roma communities survey

| Strata label | Settlements general population survey | Refusal rates (%) | Settlements Roma communities survey | Refusal rates (%) |
| --- | --- | --- | --- | --- |
| Cities > 1 mil inh | Municipiul Bucureşti | 51.3 | Municipiul Bucureşti | 35 |
| 160.000 inh. ≤ cities < 1 mil inh | Municipiul Ploieşti | 39.8 | Municipiul Ploieşti | 35 |
|  | Municipiul Craiova | 34.5 | Municipiul Craiova | 35 |
|  | Municipiul Timişoara | 38.8 | Municipiul Timişoara | 35 |
|  | Municipiul Cluj-Napoca | 36.2 | Municipiul Cluj-Napoca | 35 |
| 50.000 inh. ≤ cities <160.000 inh. | Municipiul Drobeta-Turnu Severin | 38 | Municipiul Drobeta-Turnu Severin | 35 |
|  | Municipiul Sibiu | 35 | Municipiul Sibiu | 35 |
|  | Municipiul Satu Mare | 28 | Municipiul Satu Mare | 35 |
|  | Municipiul Buzău | 47.2 | Municipiul Buzău | 35 |
| South: cities < 50.000 inh. | Oraş Plopeni | 29 | Oraş Plopeni | 35 |
| South-East: cities < 50.000 inh | Municipiul Băileşti | 17 | Municipiul Băileşti | 48 |
| West: cities < 50.000 inh | Oraş Petrila | 17 | Simeria | 35 |
| Centre: cities < 50.000 inh | Oraş Teiuş | 13.7 | Oraş Teiuş | 48 |
| North-West: cities < 50.000 inh | Oraş Năsăud | 10.2 | Oraş Năsăud | 35 |
| North-East: cities < 50.000 inh | Municipiul Fălticeni | 18.6 | Municipiul Fălticeni | 35 |
| South-East: cities < 50.000 inh | Oraş Ovidiu | 21.4 | Oraş Ovidiu | 48 |
| Bucharest-Ilfov: cities < 50.000 inh | Oraş Otopeni * | N/A * | Chitila | 35 |
| South: rural settlements | Mitreni | 0 | Mitreni | 48 |
|  | Clejani | 13.2 | Clejani | 48 |
| South-West: rural settlements | Cătunele | 23.3 | Farcaș | 35 |
|  | Giubega | 13.2 | Tânțăreni | 35 |
| West: rural settlements | Dognecea | 11.1 | Constantin Daicoviciu / Dognecea | 35 |
|  | Pietroasa | 8.3 | Checea | 35 |
| Centre: rural settlements | Rîciu | 10.8 | Rîciu | 48 |
|  | Hârseni * | N/A * | Hârseni | 48 |
| North-West: rural settlements | Ip | 11.4 | Panticeu | 35 |
|  | Ciucea | 8.3 | Românași | 35 |
| North-East: rural settlements | Şcheia | 8.1 | Sat Slobozia, Comuna Deleni | 35 |
|  | Bozieni | 11.1 | Dragomirești | 35 |
| South-East: rural settlements | Slava Cercheză | 19.5 | Foltești | 35 |
|  | Priponeşti | 10.8 | Ciucurova | 35 |
| Bucharest-Ilfov: rural settlements | Cernica | 72.9 | Cernica | 48 |

** No interviews were performed here, so refusal rates were not estimated.*

*Inh, inhabitants*
