# Supplementary material for: Differences in health-related quality of life between the Roma community and the general population in Romania
Source: J Patient Rep Outcomes. 2022 Dec 22;6:127. doi: 10.1186/s41687-022-00530-2 (PMC9780407; doi:10.1186/s41687-022-00530-2)
Supplement: Supplementary file 2 — Additional file 2. Appendix 2 – Missing data in the EQ-5D-5L measures for the Roma community (n = 687). [file 41687_2022_530_MOESM2_ESM.docx]

Appendix 2 – Missing data in the EQ-5D-5L measures for the Roma community (n=687)

| Measure | Missing (n) | Missing (%) |
| --- | --- | --- |
| Mobility | 1 | 0.15 |
| Self-Care | 1 | 0.15 |
| Usual Activities | 0 | 0 |
| Pain & Discomfort | 5 | 0.73 |
| Anxiety & Depression | 20 | 2.91 |
| EQ-VAS | 0 | 0 |
| EQ-5D-5L Index Score | 23 | 3.35 |

*EQ-VAS*, Visual Analog Scale
