# Supplementary material for: Differences in health-related quality of life between the Roma community and the general population in Romania
Source: J Patient Rep Outcomes. 2022 Dec 22;6:127. doi: 10.1186/s41687-022-00530-2 (PMC9780407; doi:10.1186/s41687-022-00530-2)
Supplement: Supplementary file 4 — Additional file 4. Appendix 4 - Differences in EQ-5D-5L utility scores between the general population and Roma respondents from Propensity Score Matching Models (n = 1589). [file 41687_2022_530_MOESM4_ESM.docx]

Appendix 4 - Differences in EQ-5D-5L utility scores between the general population and Roma respondents from Propensity Score Matching Models (n=1,589)

| Model | Roma | Lower 95% Confidence Interval | Upper 95% Confidence Interval |
| --- | --- | --- | --- |
| ATET | -0.040***  (0.011) | -0.061 | -0.020 |
| Notes: Average treatment effect on the treated (ATET) from a propensity score matching model.  Robust standard errors in parentheses. *p<0.1, **p<0.05, ***p<0.01 | | | |
